# Functional outcomes of tibiotalocalcaneal arthrodesis using a hindfoot arthrodesis nail in treating Charcot's arthropathy deformity

**DOI:** 10.3389/fsurg.2022.862133

**Published:** 2023-01-20

**Authors:** Mohd Yazid Bajuri, Ammar Muizuddin Manas, Kamarul Syarazi Zamri

**Affiliations:** Department of Orthopaedics and Traumatology, Faculty of Medicine, National University of Malaysia, Cheras, Malaysia

**Keywords:** hindfoot arthrodesis nail, Charcot’s arthropathy, functional outcome, orthopedics, foot and ankle

## Abstract

**Background:**

Tibiotalocalcaneal arthrodesis or hindfoot fusion is a salvage surgical option used to treat symptomatic or severe deformity as a result of Charcot’s arthropathy. It is an internal fixation that utilizes nails to stabilize the hindfoot after surgical correction of the deformity. This study intends to measure the change in functional outcomes of patients with Charcot's arthropathy using this technique and the time taken to achieve fusion.

**Method:**

This study presents a series of 40 cases of Charcot's arthropathy where hindfoot fusion was done using a hindfoot arthrodesis nail. A retrospective analysis was done where these patients’ functional scores had been evaluated preoperatively and postoperatively (serially) with the Short-Form Health Survey 36 (SF-36), American Orthopedic Foot and Ankle Society (AOFAS), Foot and Ankle Outcome Score (FAOS), and Foot Function Index (FFI). Along with its complication, the length of time required for the fusion is also reported.

**Results:**

This study consists of 40 patients (13 men, 27 women; mean age 60.5 years; age range 52–68 years) with a mean follow-up of 64 months (range 24–108 months). The mean time taken for fusion was 5.1 months. All patients showed improvement in functional scoring (SF-36, AOFAS, FFI, and FAOS) postoperatively. We establish that the improvements were gradual over 2 years. Approximately 37.5% of patients had a minor complication and 2.5% had a major complication.

**Conclusion:**

Hindfoot fusion using a hindfoot arthrodesis nail results in improved functional outcome with an acceptable fusion time and acceptable complication rate.

**Level of evidence:**

Level III.

## Introduction

Charcot’s arthropathy is characterized by progressive disruption of the foot and ankle's architecture due to loss of protective sensations to the periphery. This condition is considered rare, with a prevalence rate of approximately 0.1%–7.5% in the population with diabetic foot ulcers (1). However, it poses important and challenging issues to orthopedic surgeons. That is, it can cause deformity resulting in abnormal loading contact points, which can lead to the development of foot ulcers. This deformity or ulcer may cause limb-threatening conditions, such as abscess, osteomyelitis, and gas gangrene, which may require amputation.

The management of Charcot’s arthropathy should focus on reducing the destructive process of the disease by preventing abnormal loading in the foot and ankle, which may lead to poor healing and ulcer formation (2). Some patients may benefit from conservative treatment if they sought early treatment, which results in less deformity that can be managed with footwear modifications. The risk of surgery exceeds its benefit. Therefore, conservative treatment is the best course of action for high-risk patients with several comorbidities. Before considering surgical treatment, offloading treatment with Charcot restrain orthotic walker devices, use of braces, or total contact casting should be attempted conservatively. However, in advanced-stage deformity, some patients may require surgical correction to obtain a plantigrade foot and ankle to prevent ulceration and to achieve a stable foot and ankle for ambulation.

Several methods, including the use of internal and external fixators, have been used for hindfoot fusion (3). However, since Charcot’s arthropathy is a rare disease, data on its functional outcome and fusion time are limited. Hindfoot fusion has superior biomechanics properties compared with other strategies. Thus, *via* this study, we wanted to evaluate the functional outcome of this method using the Expert Hindfoot (tibiotalocalcaneal) Arthrodesis Nail in Charcot’s arthropathy. This retrospective study aimed to evaluate the functional outcome of tibiotalocalcaneal arthrodesis using hindfoot arthrodesis nails for treating deformities caused by Charcot’s arthropathy.

## Materials and methods

Patients who were diagnosed with Charcot’s arthropathy and who underwent hindfoot fusion using the Expert Hindfoot Fusion Nail (DePuy Synthes, Johnson & Johnson) at our institution from May 2013 to May 2018 were included in the analysis. This study was approved by the research ethics committee of The National University of Malaysia (UKM PPI/111/8/JEP-2019-672). All patients provided informed consent.

We selected patients with Charcot’s arthropathy on radiograph image and type II diabetes mellitus who answered a set of questionnaires [American Orthopaedic Foot & Ankle Society (AOFAS), Foot Function Index (FFI), 36-Item Short-Form Health Survey (SF-36), and Foot and Ankle Outcome Score (FAOS)] preoperatively and 6 weeks, 3 and 6 months, and 1 and 2 years postoperatively. Patients with any preoperative ulcer regardless of size, active infection, chronic osteomyelitis, large talus defect, advanced-stage talar body necrosis, and poor distal limb vascularity were excluded from the study. In total, 40 patients matched the criteria, and were included in the study.

The functional status of the foot and ankle was evaluated during the follow-up using the SF-36, AOFAS, FAOS, and FFI. The questionnaires are validated and reliable, and they are used widely in foot- and ankle-related studies worldwide.

Postoperatively, the patients were advised to visit the clinic regularly for wound dressing and monitoring. They were placed on a below-knee back slab and instructed to perform non-weightbearing ambulation until wound healing. They received regular physiotherapy for limb strengthening, and they were advised to conduct non-weightbearing ambulation at the operative site using crutches or a walking frame. Radiographic evaluation was performed during serial visits, and images were reviewed to evaluate for union of arthrodesis and signs of implant failure or infection. A single orthopedic foot and ankle consultant performed the evaluation.

Any surgical-related complications, including infection requiring prolonged antibiotic usage, and need for surgical debridement, implant removal, or any additional procedures, such as bone grafting and dynamization, were recorded. The complications were classified as minor and major. The minor complications included superficial infection requiring surgical debridement or screw removal. Meanwhile, the major complications were deep infection necessitating whole implant removal or lower-limb amputation.

Supplementary Flowchart 1 shows the process guideline of this study. Data entry and analysis were performed using IBM SPSS Statistics. The Wilcoxon signed-rank test was applied for SF-36 Physical Score, SF-36 Mental Score, AOFAS, FFI, and FAOS to determine the difference between the functional score of the ankle both pre- and postoperative of tibiotalocalcaneal arthrodesis using a hindfoot arthrodesis nail. The fusion rate was determined by performing a serial x-ray during follow-up and the union was defined based on the ASAMI scoring system (5). The sample population calculated for this study was measured using this formula:

$$n=\frac{2\sigma^{2}}{\Delta^{2}}(Z_{\alpha }+Z_{\beta }{)}^{2}$$

where *n* = number of samples, *σ *= population standard deviation (calculated from pool variance), Δ* *= difference in means, *Z_α_* = 1.96 for *α* = 0.05, *Z_β_* = 0.84 for 80% power, *σ* = √So2 = √790.42 = 28.11, Δ = difference in population means = 30.5, n = [2(28.11)2/(30.5)2] * [1.96 + 0.84]2 = 13, additional 20% of rejection = 13 + 2 = 15, and n × 2 = 30.

Therefore, the estimated total number of sample size will be 30 patients for this study. We selected 40 patients to have a better quality study.

## Results

In total, 40 patients participated in the study. Among them, 27 (67.5%) were women and 13 (32.5%) men. Their mean age was 60.5 ± 8.37 years (range 52.0–68.0 years). The number of patients whose right leg was affected (50%) was equal to that of patients whose left leg was affected (50%). All surgeries were performed during the inactive phase of Charcot’s arthropathy (stage 3 based on the Eichenholtz classification). The mean follow-up time after surgery was 64 ± 30.4 months (range 24–108 months). There were no dropouts during the observation period.

The Wilcoxon signed-rank test was used as the data were not normally distributed, as shown in Table 1, to compare the preoperative and postoperative SF-36 physical score, SF-36 mental score, AOFAS, FFI, and FAOS. The mean SF-36 score and AOFAS increased, which indicated improvement in function. Meanwhile, the mean FFI and FAOS decreased, which also indicated improvement in function. The mean and standard deviation of the functional scores during the preoperative and immediate postoperative periods and at each postoperative follow-up at 3 and 6 months and 1 and 2 years were calculated, as shown in Table 2.

**Table 1:** Test of normality.

	Kolmogorov–Smirnov^a^			Shapiro–Wilk		
	Statistic	df	Sig.	Statistic	df	Sig.
SF-36 pre Physical	0.539	40	0.000	0.234	40	0.000
SF-36 post Physical	0.538	40	0.000	0.147	40	0.000
SF-36 post 3 months Physical	0.510	40	0.000	0.369	40	0.000
SF-36 post 6 months Physical	0.138	40	0.052	0.895	40	0.001
SF-36 post 1 year Physical	0.525	40	0.000	0.191	40	0.000
SF-36 post 2 years Physical	0.538	40	0.000	0.147	40	0.000
SF-36 pre Mental	0.288	40	0.000	0.825	40	0.000
SF-36 post Mental	0.395	40	0.000	0.579	40	0.000
SF-36 post 3 months Mental	0.393	40	0.000	0.570	40	0.000
SF-36 post 6 months Mental	0.393	40	0.000	0.570	40	0.000
SF-36 post 1 year Mental	0.514	40	0.000	0.381	40	0.000
SF-36 post 2 years Mental	0.514	40	0.000	0.381	40	0.000
AOFAS pre	0.261	40	0.000	0.819	40	0.000
AOFAS post	0.406	40	0.000	0.662	40	0.000
AOFAS post 3 months	0.381	40	0.000	0.622	40	0.000
AOFAS post 6 months	0.396	40	0.000	0.588	40	0.000
AOFAS post 1year	0.254	40	0.000	0.861	40	0.000
AOFAS post 2 years	0.247	40	0.000	0.834	40	0.000
FFI pre	0.265	40	0.000	0.885	40	0.001
FFI post	0.141	40	0.042	0.941	40	0.037
FFI post 3 months	0.149	40	0.025	0.932	40	0.019
FFI post 6 months	0.129	40	0.091	0.946	40	0.054
FFI post 1 year	0.089	40	0.200^b^	0.982	40	0.753
FFI post 2 years	0.069	40	0.200^b^	0.975	40	0.504
FAOS pre	0.273	40	0.000	0.759	40	0.000
FAOS post	0.264	40	0.000	0.814	40	0.000
FAOS post 3 months	0.222	40	0.000	0.862	40	0.000
FAOS post 6 months	0.138	40	0.055	0.872	40	0.000
FAOS post 1 year	0.175	40	0.003	0.831	40	0.000
FAOS Post 2 years	0.188	40	0.001	0.807	40	0.000

SF-36, 36-Item Short-Form Health Survey; AOFAS, American Orthopaedic Foot & Ankle Society; FAOS, Foot and Ankle Outcome Score.

^a^ Lilliefors significance correction.

^b^ This is a lower bound of the true significance.

**Table 2:** The mean and standard deviation value of each functional scoring from preoperative, immediate postoperative, and postoperative 3 months, 6 months, 1 year, and 2 years.

Follow-up	SF-36 Physical mean (SD)	SF-36 Mental mean (SD)	AOFAS mean (SD)	FFI mean (SD)	FAOS mean (SD)
Preoperative	2.25 (9.997) *p* = 0.180	42.1 (22.188) *p* = 0.000	16.28 (10.158) *p* = 0.000	79.65 (9.085) *p* = 0.000	58.93 (12.901) *p* = 0.000
Postoperative	0.25 (1.581) *p* = 0.222	94.4^a^ (10.975) *p* = 0.000	65.18^a^ (10.698) *p* = 0.000	77.38^a^ (9.339) *p* = 0.000	55.40^a^ (11.659) *p* = 0.000
Postoperative 3 months	5.25^a^ (15.189) *p* = 0.000	94.5^a^ (10.896) *p* = 0.000	66.40^a^ (8.491) *p* = 0.000	67.62^a^ (9.737) *p* = 0.000	47.23^a^ (10.939) *p* = 0.000
Postoperative 6 months	59.15^a^ (12.608) *p* = 0.000	94.5^a^ (10.896) *p* = 0.000	66.60^a^ (8.381) *p* = 0.000	37.03^a^ (4.979) *p* = 0.000	35.85^a^ (9.963) *p* = 0.000
Postoperative 1 year	97.88^a^ (11.26) *p* = 0.000	98.7^a^ (3.667) *p* = 0.000	83.65^a^ (4.748) *p* = 0.000	21.55^a^ (5.565) *p* = 0.000	29.05^a^ (10.117) *p* = 0.000
Postoperative 2 years	98.25^a^ (11.068) *p* = 0.000	98.7^a^ (3.667) *p* = 0.000	84.10^a^ (4.119) *p* = 0.000	16.32^a^ (8.166) *p* = 0.000	25.45^a^ (10.008) *p* = 0.000

AOFAS, American Orthopaedic Foot & Ankle Society; FFI, Foot Function Index; SF-36, 36-Item Short-Form Health Survey; FAOS, Foot and Ankle Outcome Score.

^a^ Significant value *p* < 0.001.

This test revealed that the data were not normally distributed. The Wilcoxon signed-rank test was employed in view of this.

**Figure 1:**
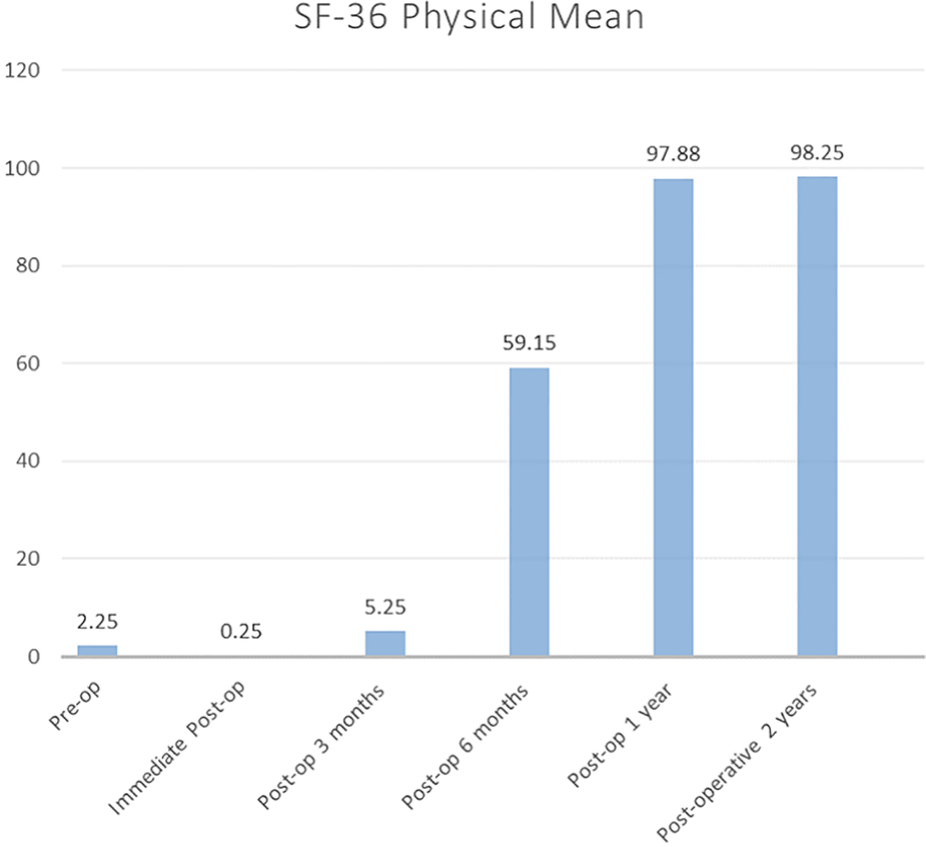
SF-36 physical component mean score for 2 years follow-up. SF-36, 36-Item Short-Form Health Survey.

**Figure 2:**
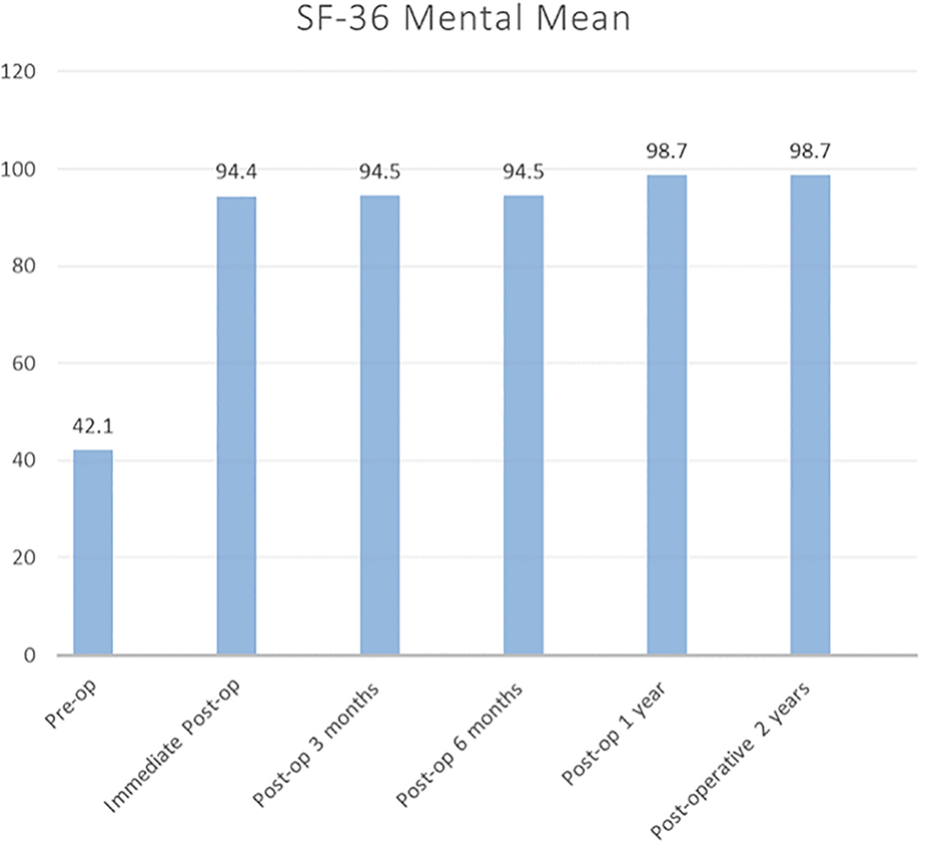
SF-36 mental component mean score for 2 years follow-up. SF-36, 36-Item Short-Form Health Survey.

Figure 1 shows the SF-36 physical score. There was minimal improvement noted from the postoperative phase up to 3 months of follow-up. The mean score improved from 5.25 to 59.15. Therefore, the patients had significant improvement at 6 months onward. The score continually improved to 97.88 until 1 year. Then, it almost plateaued, and a similar outcome was observed at 2 years. During the postoperative stage, the mean SF-36 mental score immediately improved from 42.1 to 94.4. As depicted in Figure 2, this plateaued from there onward until 2 years.

**Figure 3:**
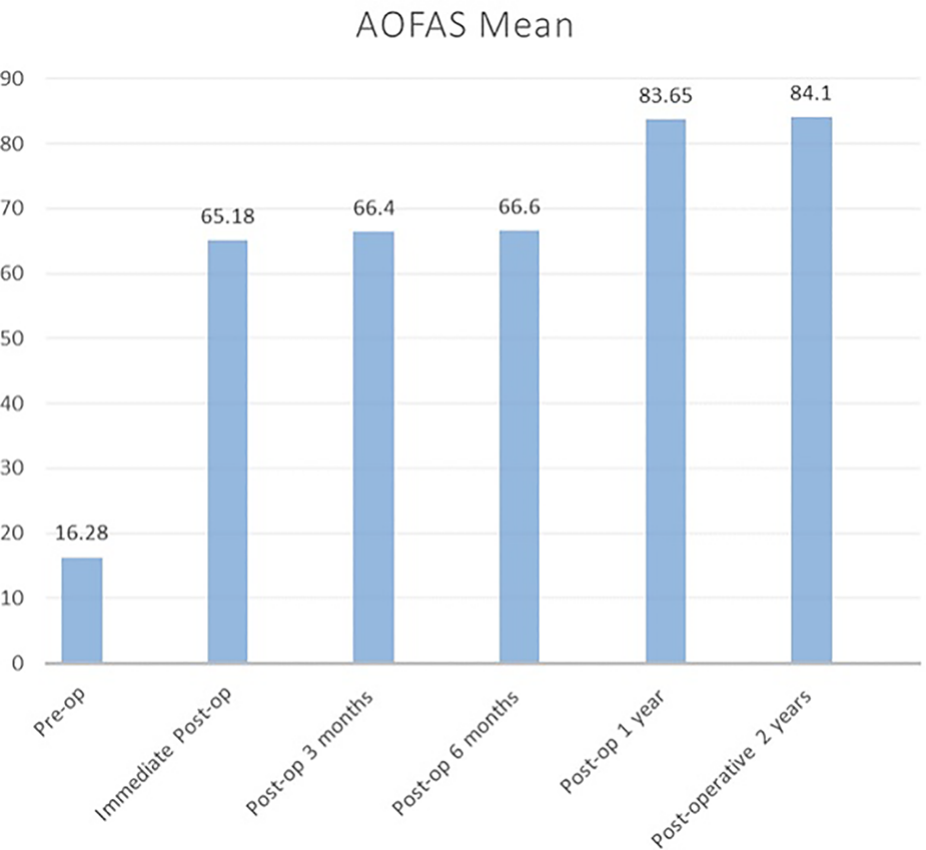
AOFAS mean score for 2 years follow-up. AOFAS, American Orthopaedic Foot & Ankle Society.

**Figure 4:**
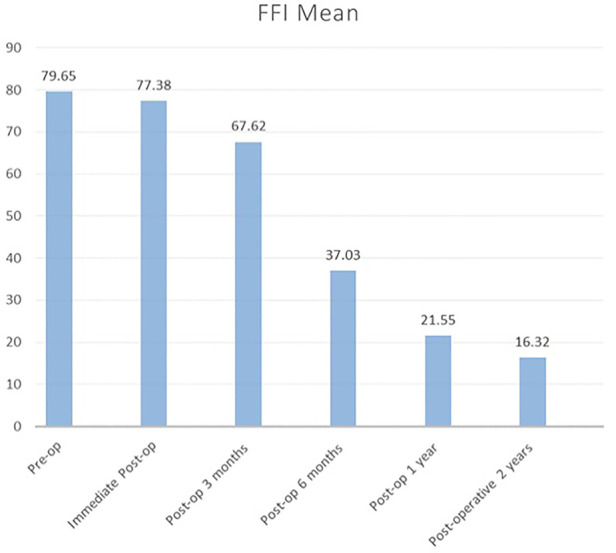
FFI mean score for 2 years follow-up. FFI, Foot Function Index.

The AOFAS score immediately improved postoperatively from 16.28 to 65.18, and improvement gradually increased to 83.6 until 1 year postoperatively. Then, it plateaued until 2 years, as depicted in Figure 3. Moreover, as shown in Figure 4, the mean FFI score decreased, which indicated gradual improvement in function initially from the preoperative stage (79.65) to 3 months postoperatively (67.62). The improvement was significant at 6 months (37.03) until 2 years (16.32).

**Figure 5:**
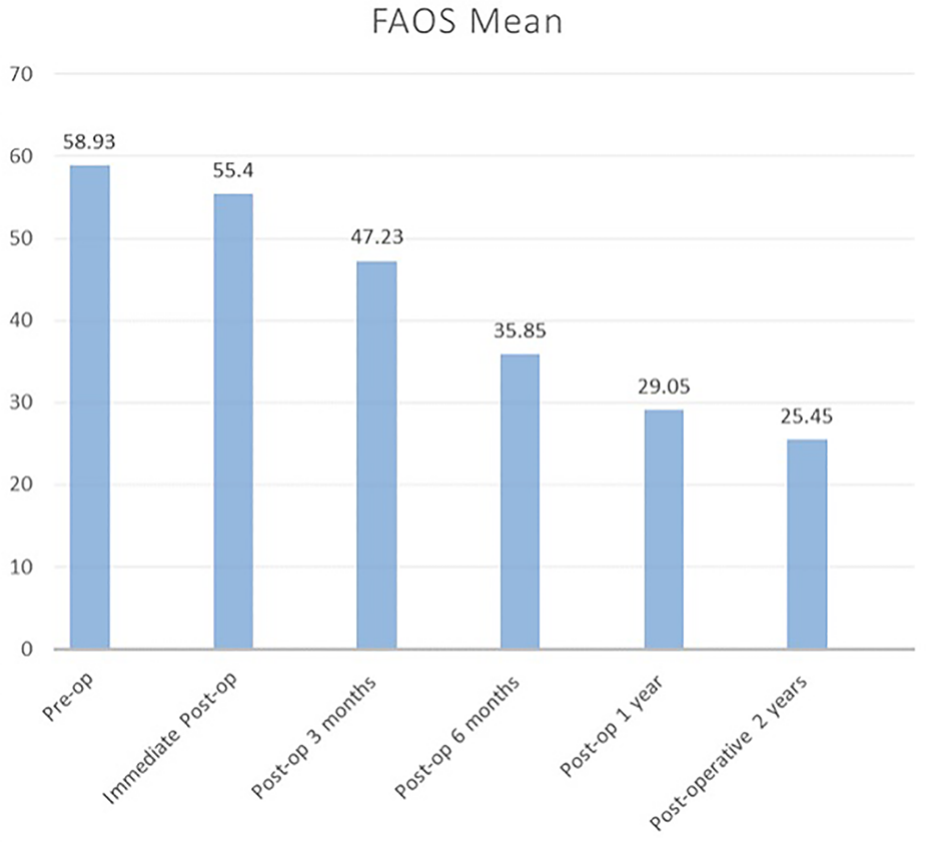
FAOS mean score for 2 years follow-up. FAOS, Foot and Ankle Outcome Score.

Figure 5 shows the FAOS score. The patients presented with a similar decrease in scores, which indicated improvement in functional outcome. Initially, the score gradually improved from 58.93 to 47.2 from the preoperative phase to 3 months postoperatively. Further, it was more remarkable at 6 months postoperatively (35.85) up to 2 years postoperatively (25.45).

All patients achieved fusion based on the radiological report. The mean fusion time was 5.1 ± 2.2 months (range 3–10 months). All limbs (100%) were saved, and amputation was not required.

However, 19 (45%) patients had an infection, which required prolonged ward admission for antibiotic treatment. Among them, 15 (37.5%) underwent wound debridement and/or screw removal. Patients who required surgery had an average of two surgeries in the operating room.

**Figure 6:**
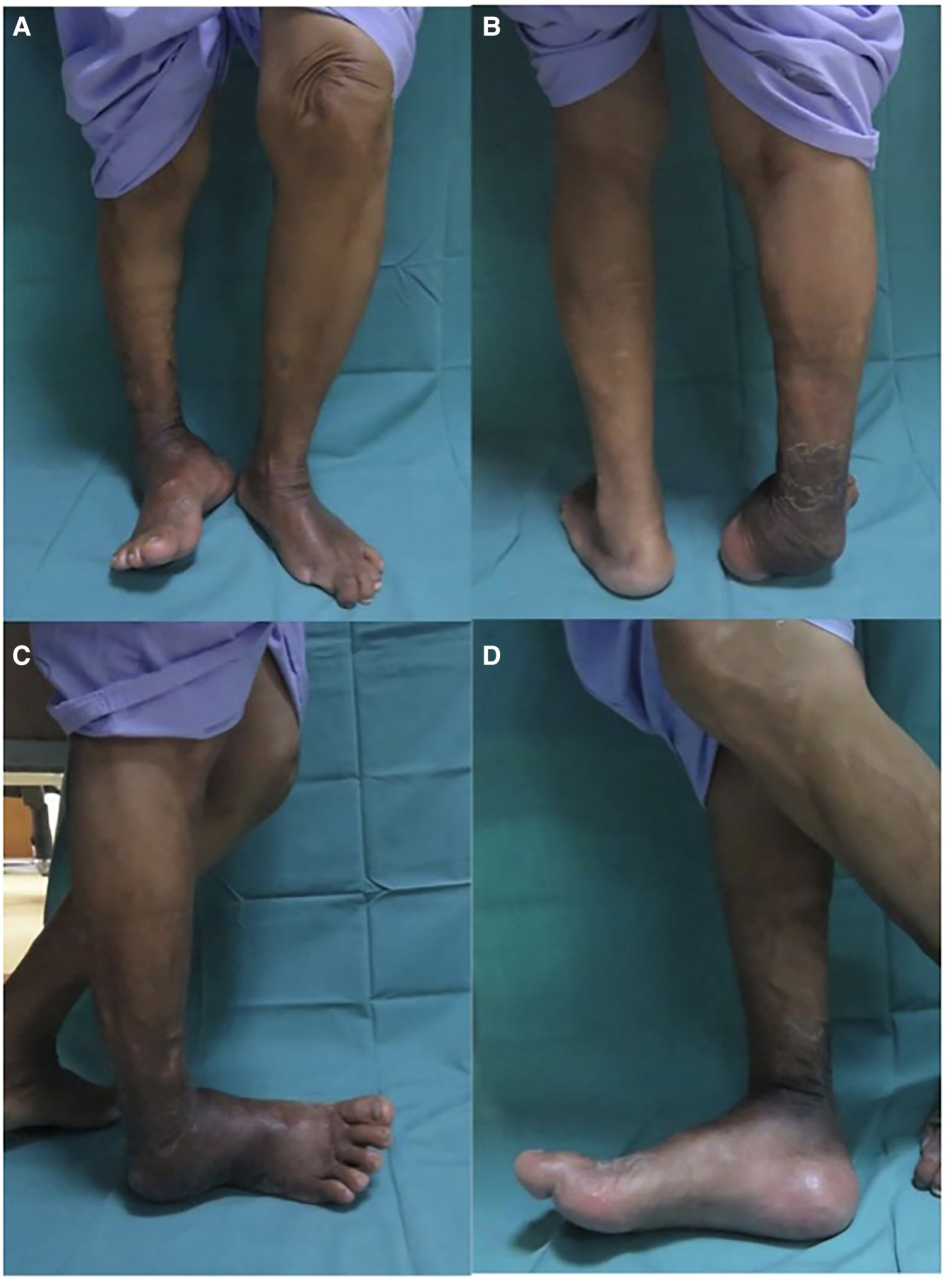
(**A**) Example clinical pictures of one of the patient's ankle preoperatively (**A**) anterior view, (**B**) posterior view, (**C**) lateral view, and (**D**) medial view. (**A,B**)Varus deformity of the hindfoot which lead to abnormal weightbearing area. (**C,D**) Patient weightbearing on lateral aspect of the foot.

**Figure 7:**
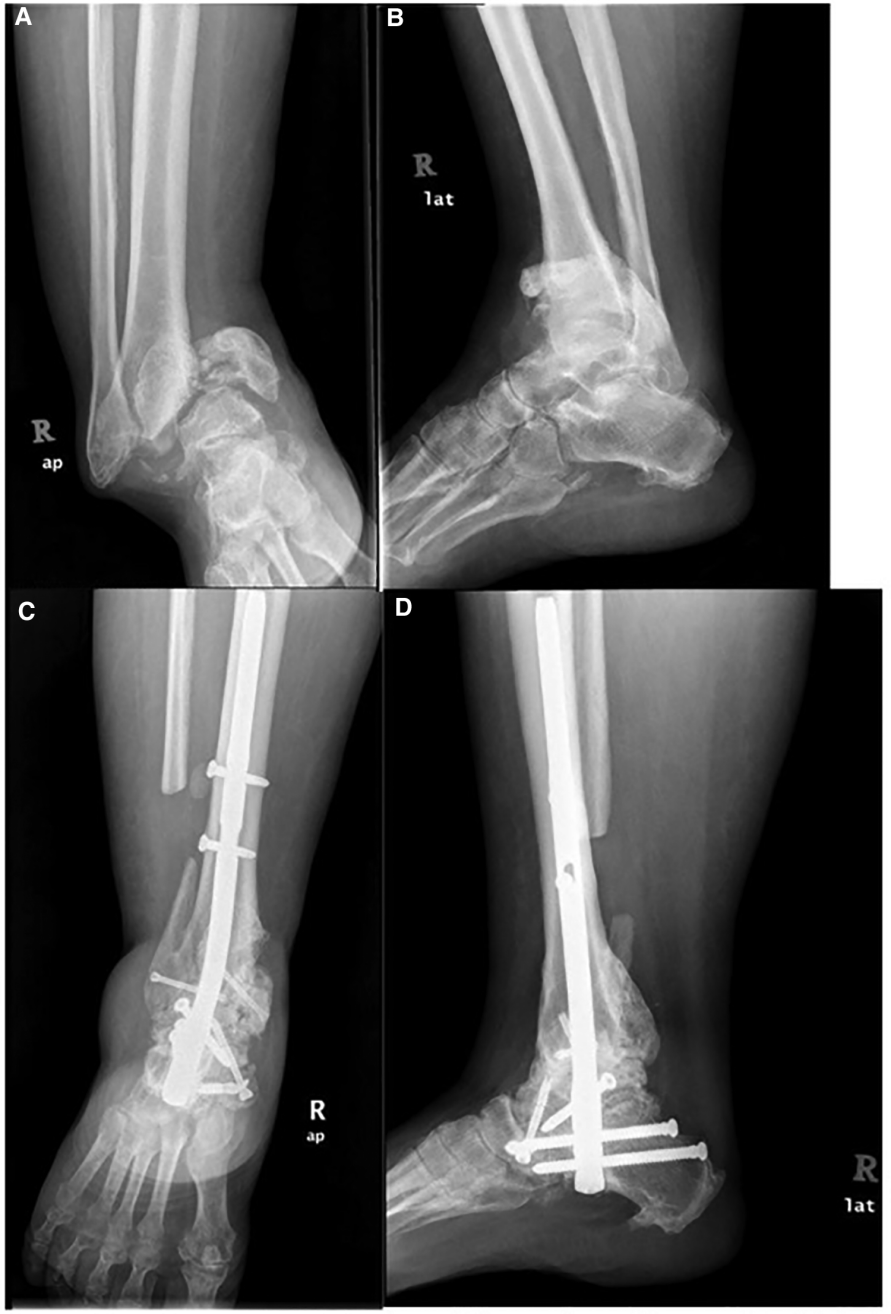
Illustrate anteroposterior (AP) and lateral radiograph of right ankle preoperative and 6 months postoperative of the same patient. (**A**) AP and (**B**) lateral radiograph shows severe ankle dislocation and medial malleolus hinging on proximal tibia cortex with consolidation of fragments indicating Eichenholtz stage 3. (**C**) AP and (**D**) lateral radiograph shows correction of deformity in plantigrade position and fused ankle joint.

Only 1 (2.5%) patient had a major complication, which required the removal of the whole implant due to severe peri-implant infection. Initially, fusion occurred at 5 months. However, 18 months postoperatively, the patient had a peri-implant infection. After serial wound debridement and antibiotic therapy, the infection was still persistent, and the nail had to be removed. After removal, the patient was allowed to perform gradual weightbearing ambulation, and the infection eventually subsided. Figure 6 shows the clinical images of an ankle preoperatively. As depicted in Figures 6A,B, this patient had varus deformity of the hindfoot, which led to abnormal weight bearing. Figures 6C,D present weightbearing on the lateral aspect of the foot. Figure 7 shows the anteroposterior and lateral radiograph images of the right ankle preoperatively and 6 months postoperatively in the same patient. Preoperatively, severe ankle dislocation and medial malleolus hinging on the proximal tibia cortex with consolidation of fragments were observed. These indicated stage 3 Charcot’s arthropathy based on the Eichenholtz classification (Figures 7A,B). At 6 months postoperatively, deformity in the plantigrade position and fused hindfoot was noted (Figures 7C,D). One patient was treated successfully using a hindfoot arthrodesis nail.

## Discussion

Currently, there are several studies on the functional outcomes of hindfoot fusion using different methods, such as the application of an external fixator, hindfoot nailing, and plating in different conditions, including post-traumatic ankle osteoarthritis, Charcot’s arthropathy, and avascular necrosis (3). However, Charcot’s arthropathy should be evaluated as a separate group for hindfoot fusion. This is because patients with this condition are commonly immunocompromised because of diabetes mellitus, have poor bone quality, and are older with multiple comorbidities than those with post-traumatic ankle osteoarthritis (6). To date, there are only eight studies evaluating the outcome of hindfoot arthrodesis using intramedullary nails in Charcot’s arthropathy (7).

According to Brodsky et al., the midtarsal (60%) is the most common site of a Charcot deformity, followed by the tibiotalar joint (20%), subtalar joint (10%), combination of areas (<10%), and forefoot alone (<10%) (8). However, the ankle is less commonly involved. However, if it is, progressive deformity and loss of function of the lower limb, such as that in the varus and valgus of the ankle, commonly occur. If such a deformity is left untreated, it will lead to abnormally high pressure on the medial or lateral side of the foot and ankle during ambulation. This is coupled with a loss of neuroprotective sensation to the lower limb, which can be a predisposing factor of ulcer development and infection.

The management of ulcers is challenging due to persistent abnormal weightbearing. If an ulcer is complicated with infection, the levels of infective markers, such as high-sensitivity C-reactive protein, procalcitonin, and white blood cells, will increase (9). Its treatment includes empirical antimicrobial therapy, followed by definitive antimicrobial therapy after culture and sensitivity testing. Nevertheless, in some cases, surgical intervention, including drainage and debridement and osseous resection in the presence of osteomyelitis, may be required (10). In addition, hyperbaric oxygen therapy was found to be an effective adjunct modality for chronic diabetic foot infections by promoting faster wound healing (11). Despite several new modalities for managing ulcers, such as advanced dressing, antimicrobial dressing, negative pressure dressing, and hyperbaric oxygen therapy, numerous patients have reported a significant reduction in quality of life with the management of diabetic foot ulcer (12). Due to progressive ankle deformity, challenges in ulcer management, reduced quality of life, and, occasionally, lower-limb infection, some patients required major amputation. The rate of amputation is approximately 4.5% in Charcot’s arthropathy without an ulcer, and it is even worse in Charcot’s arthropathy with an ulcer, with an incidence of as high as 21.3%, which is about one in five individuals (13).

Nevertheless, deciding for a major amputation is not an easy task as the procedure is associated with not only morbidity, such as physical pain and increased energy consumption for ambulation, but also an increased mortality risk. According to a systematic review conducted in 2016, major amputation has a significantly high mortality rate. That is, the 5-year mortality rates are in the range of 40%–82% for transtibial amputation and 40%–90% for transfemoral amputation (14).

Therefore, currently, several foot and ankle surgeons are going toward a limb-salvage procedure with the correction of deformity and fusion to obtain a stable, plantigrade foot and ankle. According to Gil et al., the cost of a limb-salvage procedure is almost similar to that of a limb amputation in a series of 93 patients. The study was conducted over a 12-month period and included inpatient hospitalization, rehabilitation or admission to a nursing facility, and the purchase of a prosthetic or appropriate footwear (15). It is worth selecting patients who must undergo a limb-salvage procedure with consideration of the current morbidity rate, wound condition, baseline infective marker levels, diabetic control, and patient demand. Thus, their limbs could be saved, and they can ambulate without using a walking aid or prosthesis.

The ideal timing of surgery is still controversial, and previous studies have different results. It varies between foot and ankle surgeons, and there is no universally accepted guideline or definite algorithm. Traditionally, the acute stage (fragmentation phase) is a relative contraindication to arthrodesis. The main concern at this phase is the potential delay in wound healing or surgical site infection because of limb edema. More importantly, due to osteoclastic resorption of the bone in this phase, it could potentially cause hardware loosening, delayed union, or nonunion (16).

Nevertheless, there are reports about surgeons performing arthrodesis in the fragmentation phase when there is collapse and deformity. Simon et al. has the most cited article advocating surgical intervention. In that study, 14 patients had arthrodesis (17). These patients remained non-weightbearing until there is evidence of consolidation on radiography. Then, the management strategy was changed to an assisted short leg cast followed by a nonassisted short leg cast. The mean time to return to normal shoes and full weightbearing was 27 weeks, and no complications were observed at a mean follow-up of 41 months. A potential ulcer is a cause of concern in early surgery if further deformity occurs and there are difficulties associated with prolonged immobilization with contact casting.

However, the time to return to normal shoes (27 weeks) in the previous study is almost the same as the duration of conservative treatment. Armstrong et al. showed that the mean duration of conservative management before returning to normal shoe and full weightbearing in acute Charcot’s arthropathy was 26 weeks (18). Therefore, if surgery should be performed in the acute phase, the indication could be a cause of concern for skin breakdown, severe dislocation, and severe instability, or conservative management failed to obtain a plantigrade foot (16). Some patients may benefit from conservative treatment if they sought early treatment, thereby resulting in less deformity that can be managed via the use of modified footwear. Conservative treatment is the best option for high-risk patients with multiple comorbidities because the risks of surgery outweigh its benefits.

By contrast, some surgeons prefer to perform surgery in the nonacute phase of Charcot’s arthropathy (stage 2 or 3). This is to prevent surgery in the edematous limb and during the osteoclastic phase of the disease. Some argue that the best time to do the surgery is at stage 2 as the deformity is still easily reducible and surgical correction is relatively simpler to perform.

Silvampatty et al. evaluated 33 patients with Charcot’s arthropathy who underwent hindfoot arthrodesis at different stages (from stage 1 to 3). Results showed no significant difference in the functional scoring of the hindfoot and complication rate after a mean period of 40 months (19). Previous studies on hindfoot fusion in Charcot’s arthropathy are commonly retrospective in nature, and they only included a small number of patients. Hence, regardless of these factors, the latest systematic review has revealed that the timing of surgery remains unclear (20). As in this study, all patients underwent surgery at the consolidation and remodeling phase (stage 3). The incidence of complications is lower, the functional outcome is good, and there is no risk of implant loosening.

There are several fixation methods for correcting hindfoot deformity via either external or internal fixation. These include the use of Steinmann pins, screws, plates, angle blade plates, ring fixator, and intramedullary nails (21). All these strategies aim to achieve a stable hindfoot while fusion takes place. However, notably, Charcot’s arthropathy is associated with poor bone quality and soft tissue coverage issues, such as ulcers and skin dryness. Therefore, selecting the type of implant is more complicated.

In our study, all patients used the same implant, and the procedures were performed by a single surgeon to minimize bias. Our patients underwent internal fixation as they were all in the nonacute phase (stage 3) and had no ulcerations. We opted to use a hindfoot nail for our fusion technique as it has more superior biomechanical properties to be used in relatively osteopenic bone quality in patients with Charcot’s arthropathy. A biomechanical study has shown that intramedullary nailing has increased rotational stability, bending stiffness, and dynamic compression capability compared with lag screws, external fixator, and plates (22, 23).

In hindfoot Charcot’s arthropathy, there is more likely a large bone defect particularly in an extremely deformed ankle. This poses a challenge to surgeons during arthrodesis due to the need to use a bone allograft or autograft of variable sizes to fill the defect area. Using a bone graft, we added a bone interface that should heal, and there is a risk of structural collapse of the hindfoot due to slow or non-incorporation of the bone graft or even a risk of infection. According to the most recent systematic review, the rate of infection for hindfoot arthrodesis is in the range of 11%–85% (4). In our case, it was approximately 37.5%. Only minor complications were observed, and limb amputation was not performed.

All patients (100%) achieved union. In the literature, the union rate for hindfoot fusion using allograft or autograft is 58%–93% (22, 24). In our study, the mean fusion time is 5.1 months, which is slightly better than that of other studies (6–10 months) that used hindfoot arthrodesis nails (19, 25, 26).

The functional outcome of the hindfoot was gradual. The improvement in AOFAS and SF-36 mental scores can be observed in the immediate postoperative period, and it gradually increased. However, the FFI, FAOS, and SF-36 physical scores significantly improved at 6 months onward. This could be attributed to the fact that the mean fusion time is 5.1 months, which marks the period when patients are allowed to completely bear weight. The limitation of the current study is that the sample size was not large enough. Hence, the results might not be generalizable.

## Conclusion

Charcot’s arthropathy is a rare condition. However, orthopedic surgeons find it important as it poses a threat to the affected limb. This condition should be treated early with bracing or casting and surgery to correct the deformity, which is occasionally required to prevent ulcers caused by nonuniform loading in the foot and ankle. Hindfoot fusion using hindfoot arthrodesis nails can gradually improve the hindfoot functional outcome within 2 years with comparable fusion time to other studies and with a minimal complication rate. Therefore, hindfoot fusion with hindfoot arthrodesis nails has superior biomechanics properties compared with conventional methods. Hence, it is strongly recommended for Charcot’s arthropathy. Further, it is an excellent technique in limb salvage, has a good fusion rate, and can prevent major complications.

## Data Availability

The original contributions presented in the study are included in the article/Supplementary Material, further inquiries can be directed to the corresponding author.
