# Supplementary figures and images for: Functional outcomes of tibiotalocalcaneal arthrodesis using a hindfoot arthrodesis nail in treating Charcot's arthropathy deformity
Source: Front Surg. 2023 Jan 20;9:862133. doi: 10.3389/fsurg.2022.862133 (PMC9895943; doi:10.3389/fsurg.2022.862133)

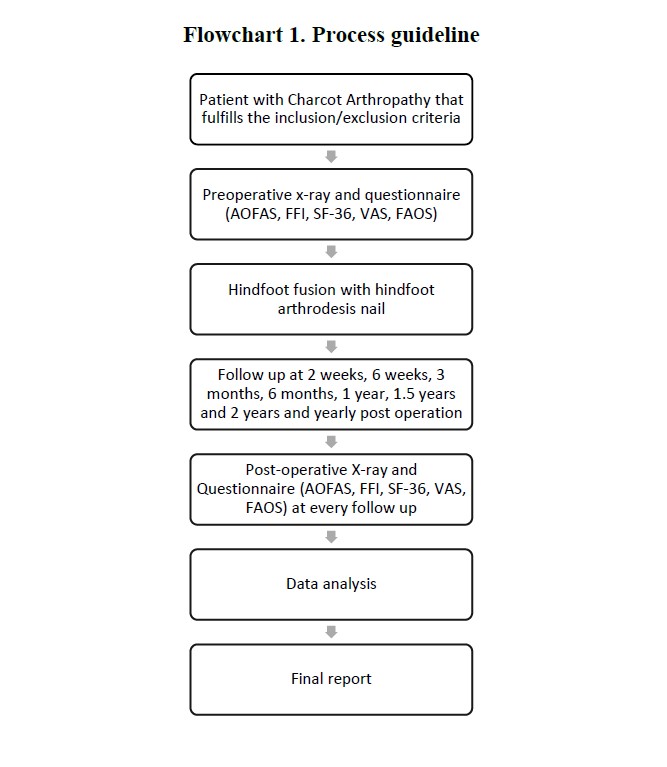

Supplement: Supplementary file 1 [file Image1.jpeg]
